# Traveling Letters from the Senior Editor

**Published:** 1847-10

**Authors:** 


### THE WESTERN JOURNAL.

New Series.—Vol. VIII.—No. IV.

LOUISVILLE, OCTOBER 1, 1847.

### TRAVELING LETTERS FROM THE SENIOR EDITOR.

NUMBER THREE.

*Chautauque Lake, N. Y.,*
August 1st, 1847.

*To My Colleagues:*

Gentlemen:—The beautiful miniature lake, on the banks of which I am now writing, is 1291 feet above the sea, and 700 feet above the average level of the "great lakes," from Erie to Superior,—thus defect in area, is compensated by loftier elevation. For several years there was on this mountain water, a steamboat which made daily trips between Jamestown and Mayville, at its opposite extremities; and, full of "human nature," I promised myself a voyage on the highest level which has yet been navigated by steam in our own or any other country, but on arriving, I found the little steamer with her fires extinguished, because of deficient patronage. We, who love to claim for our beloved Ohio all that justice will permit, and perhaps a little more, may write down and publish, that Chautauque lake, although within 80 miles of the Falls of Niagara, prefers the "sunny south," and pours its superfluous waters into the Allegheny. This it does by sending them through the obsolete bed of another lake of similar size, which has been permanently drained into that river at the beautiful mountain village of Warren, in the State of Pennsylvania, where my last letter was written.

The gently rolling table land around the lake is about two or three hundred feet above its surface, and being fertile, well timbered, and abounding in springs, is thickly inhabited; and thus presents the best opportunities for studying the influence of altitude on health, that are, at present, to be found in the central valley of North America. In fact, if we divide the population of the valley into fifteen parts, we shall, I think, find that five live on or below the level of five hundred feet; eight within the next zone of five hundred feet; leaving not more than two for the zone from a thousand to fifteen hundred feet, the summit level of the south-west corner of the State of New York, where I now am. As we advance from south to north we know that some diseases diminish in frequency and violence, or cease altogether; while others increase in prevalence or appear for the first time. Now, is the law of higher elevation coincident with that of higher latitude in these respects? I shall not at present discuss this question of Medical Geography, for the elucidation of which I have collected several facts, but hope that some of our correspondents will favor us with their observations.

The table land, which stretches east and west from Chautauque lake, has a definite geological character. Immediately to the south, is the final out-crop of the conglomerate, which underlies the great coal formation, that extends along the western flanks of the Allegheny mountains to Alabama. Every where this conglomerate gives us high and rugged acuminate or conical hills; and every where the Devonian Sandstone and Shale, which succeed to it, present soft and gentle aspects. It is this formation which constitutes the Chautauque platform. Entirely destitute of calcareous beds, all the water would be soft, but for the great deposit of *drift*, which is not only accumulated in terraces in all these elevated valleys, but overspreads the highest ridges. Thus, at the altitude of 1600 feet, or twice the average elevation of Kentucky and Indiana, we find sand, rolled pebbles, boulders, and immense blocks, such as everywhere abound at a lower level north of the Ohio, but are no where found south of that stream. When examined, this *debris* is found to have been brought from the north; and, as to the erratic masses of granite and other primitive rocks, they are supposed by the New York geologists—or at least by Mr. Hall—to have been transported from the Adirondack mountains, west of Lake Champlain, which are more than three times as high as this table land, and were consequently two thousand feet above the level of the sea, when it was a thousand feet beneath the surface. In the valley of the Allegheny river, at Pittsburgh, and wherever I traveled in it, up to this place, I met with *drift*, but in no portion of the route, was it so abundant as in the bed of the dried up lake, through which Chautauque discharges its superabundant waters. This ancient valley, from one to four miles in width, enters that of the Allegheny by a broad opening which abounds in terraces of *drift*. Hence, it was one of the gateways, through which the traveling fragments were rolled into that valley, and cast upon the flanks of its rugged hills. A word more on this subject and I will "let you off." If the mountains of New York sent over the west huge masses of their rocky strata, it may be asked why the mountains of Virginia and North Carolina did not overspread Kentucky and Tennessee with similar blocks? Meteorology, I presume, can give a correct reply. The Adirondacks are found in the latitude of 44°—the high mountains of the south lie in 35° or 36°. The cold of the former might be sufficient to produce glaciers—that of the latter insufficient; and hence we derive an additional argument in support of the theory, which ascribes the transportation of erratic blocks to floating icebergs. I am bound, however, to mention an objection to this theory, which, I believe, has not yet been published, viz:—that these masses of rock are no *colder* than any other stones; and this reminds me of a kindred objection, by another person, to a palæontological conclusion:—Having found a *Favosite* in fresh plowed ground, he declared it was not, as the geologists believe, a product of the sea, but a petrified bee's comb for he could smell the honey!

GENESEO, N. Y., August 5th, 1847.

On leaving Chautauque Lake, and passing from the county of that name, my way was to the north-east. In the adjoining counties of Cattaraugus and Allegheny, I traversed, within a few hours, streams which fall into the Allegheny river, Lake Erie, and Lake Ontario, coming very near to others which flow into the Susquehanna. Thus I passed through a hydrographical axis, from which different parts of the same thunder shower flow to the Atlantic Ocean by the Gulf of Mexico, the Gulf of St. Lawrence and Chesapeake Bay, through 20° of latitude, and 30° of longitude. Verily, our hydrography is like our Anglo-Saxon self-complacency, on a large scale. Having traversed this centre, I came fairly into the basin of Lake Ontario, and made a somewhat rapid descent into the celebrated Genesee flats. These flats are nothing more nor less than the bottom of a drained lake, through which the Genesee river, about the size of "Salt River" (to which I refer on account of its notoriety), flows into Lake Ontario. The Genesee does not enter at the head of the old lacustrine valley, but on its western side; coming down from the Chautauque summit level, like a true mountain stream, by a series of remarkable cataracts, but flowing afterwards with a sluggish current. The town in which I am writing, is seated on the eastern bank of the valley, about seven miles below the influx of the river. For many years after the commencement of settlement, this valley, one of the most fertile in the United States, and from two to five miles wide, was infested with autumnal fever, some cases of which were as malignant as any we still meet with 10° further south. Cultivation, however, has nearly eradicated their cause, notwithstanding many portions are liable to annual inundation. Such, in reference to intermittent and remittent fever, is the favoring influence of a colder climate. It is worthy of notice that this valley, especially the part near which I am writing, was formerly inhabited by the Senecas and other tribes of the Iroquois Confederacy. Now as the Indian is by no means exempt from autumnal fever when exposed to its cause, we may infer that the disease did not prevail while the flats were covered with forest; but when they were denuded of trees, and the soil was broken up, that fever was for a time extremely prevalent.

I find in this locality a great deal of goitre, and have been told of two or three congenital cases.

The formation is Devonian shale, which crops from beneath the sandstone of the Chautauque summit level. This shale is the equivalent of that which has a line of bearing across the State of Ohio, from the mouth of the Sciota to that of the Cayuhoga. In the whole of that zone goitre prevails; and although I have met with it under different geological circumstances, I have certainly seen more of it in connexion with that formation than any other.

Speaking of geological formations leads me to speak of Geological Reports of which I have this evening made a purchase, designed for the Library of our University, if the committee of augmentation see fit to take them. They are the Reports of the Geologists, Mineralogists, Botanists, and Zoologists of the State of New York, in thirteen quarto volumes, with a separate geological map, and the *prëemption right* to two unpublished volumes—one on Agriculture, and the other on Palæontology. This work, it is said, has cost more than $400,000! What a noble example! How deserving of imitation by all the States of the Union which have not yet undertaken such an enterprize! Truly this *is* the Empire State; and yet I am sorry to be obliged to add, that imperial Republics, like Emperors themselves, may do very little as well as very great things. A short time since, it seems, the members of the legislature undertook to present themselves with copies of this magnificent work, for which, by the way, the world was indebted not to *them*, but their predecessors; but a constitutional obstacle was thrown in their path. It was, however, soon surmounted by the happy thought of selling off the work at the nominal price of a dollar a volume! The law provided, that copies should be presented to certain county academies, but passed by the colleges of the State generally, and directed that the remainder should be sent to the treasurers of the different counties, in numbers proportionate to their population, there to be sold to those who should first hand in their names. The consequence was, that a large portion of the whole edition fell into the hands of persons who cannot read them, while hundreds of scientific men and institutions, are left without any, or can only obtain them at an immense advance on the price given by speculators. Thus I was obliged to pay $50 for what the man of whom I purchased had paid only $13 the day before.

Avon Springs, August 6th.

This is the land of buggies as well as of humbuggies. Having yesterday submitted myself to one of the latter, I this morning committed myself to one of the former, and left Geneseo to spend a few hours at this watering place, and be ready for the afternoon stage to Rochester. The village of Avon, like the other villages of New York, *abounds* in white frame houses, built in good taste, and delightfully embowered in trees and flowering shrubs. The Springs boil up at the margin of the flats, and one of them is so copious, as to turn a small overshot wheel, which works a pump, whereby a quantity of the water is raised fifteen or twenty feet to form shower-baths. Such is the labor saving ingenuity of our brethren in this northern section of the Great Central Valley. The water is likewise heated in close boilers, so that invalids may have baths of every temperature, from 48° to any higher point, according to their tastes or necessities. Stately and overshadowing elms, with comfortable pavilions, are at hand, and altogether, the place has a fine appearance of comfort. But I must come to the springs themselves. They are three or four in number, and vary somewhat in their composition and effects; which are pointed out in a well written pamphlet, by Dr. Salisbury, an intelligent and and respectable physician of the village, a copy of which has just been given me. The chief gaseous ingredients are sulphuretted hydrogen and carbonic acid; but some of the fountains also contain nitrogen gas. The active saline elements are the sulphates of soda and magnesia, and the muriates of soda and lime. The sulphate and carbonate of lime are also present, though perhaps comparatively inert. The analyses were made by Professor Beck, Professor Hadley, and Dr. Chilton, of the city of New York. Waters of such a chemical constitution cannot fail to be medicinal. According to Dr. Salisbury who has observed their effects for twelve or fifteen years, they are decidedly curative, in the following maladies:—1st. Almost all chronic diseases of the skin. 2d. Torpor of the liver and dyspeptic derangements of the stomach, with constipation. 3d. Chronic inflammations of the urinary organs 4th. Chronic bronchitis. 5th. Chronic rheumatism.

Now you may ask, of what use is this account of a watering place, in the Genesee flats, to the readers of the Journal down in the south-west? I answer, a great deal. The south-west, as far as I know, can boast of no better springs; and invalids need distant excursions, a northern climate, and interesting scenes and objects. All these they will have by making their way to the lakes and traversing Erie to the Falls of Niagara, from whence in a single day, if they choose, they may, without fatigue, seat themselves under the quiet and wide-spreading elms of Avon, in as high a lititude as Saratoga. They will here meet chiefly with invalids, and be free from the annoyance of those frivolous and fashionable people, who visit more noted watering places for the purpose of displaying their accomplishments, and entrapping each other into matrimony.

Rochester, August 9th.

From Avon to this place, the distance is twenty miles, and may be traveled either by land or water, which I mention for the edification of invalids. In passing from Geneseo to Rochester—from south to north—we traverse in its shortest diameter, the zone, which Dr. Emmons, in his Report on Geology applied to Agriculture, calls the Wheat District of New York. He thinks, I believe, that something in the soil, gives to the wheat grown upon this belt, a better quality than that raised elsewhere. Certain it is that the Genesee flour maintains a very high reputation.

In descending from the hill country to this place, through a thousand feet, I have found that the lower level has much more than compensated for the higher latitude. Peaches are successfully cultivated here; while a degree further south, on the Chautauque platform, they are entirely neglected. When I was there, many fields of Indian corn were materially injured by the frost, while, in the flats, they escaped, and are, moreover, much further developed. Finally, those men who swing the scythe and cradle as a profession, after having cut the harvests below, were ascending to the south for the same object.

Rochester, with its 30,000 inhabitants, is the wonder of this region, as Cincinnati and St. Louis are of the west. Its remarkable growth is to be ascribed chiefly to the Falls of the Genesee river, the waters of which in finding the level of Lake Ontario descend, within the limits of the town, 260 feet!

It is a sort of popular opinion, both in and out of the profession, that rivers which flow through alluvial grounds absorb miasms which they give out under the agitation of rapids or cataracts. The Genesee flows for forty or fifty miles in such a bed, but miasmatic fevers are rare at Rochester in latter years, while they still prevail along the great waters of the estuary below, quite down to the Lake, the shores of which are swampy. It is a current belief, at this as in many other towns, that as autumnal fever has diminished, consumption has increased. However this may be, that fatal malady does its work most unrelentingly here, for in 1845, of 364 deaths, 77, or more than a fifth, were from that disease.

Rochester has a public cemetery called Mount Hope, which does equal honor to her taste and moral feeling. The site is a portion of the clay and gravel hills which, south of the town, range parallel to the lake, and were heaped up by its waves when they once occupied a much higher level than at present. The inequalities of surface are truly remarkable, presenting ridges, winding valleys, high mounds and funnel-shaped depressions of great depth. Nearly the whole is covered with native forest trees and shrubs, with which exotic flowers, both perennial and annual, bloom in striking contrast. The deep and dark pits are terraced to the very bottom, and converted into sepulchral craters, which in the dim light of the evening sun display white monuments that stand like guardian angels over the remains of the dead. From the highest eminence the blue line of Lake Ontario appears in the distance, while from other parts the Genesee river is seen like a moat protecting the consecrated grounds from intrusion. Paths and carriage roads conforming to all inequalities of surface carry the visitor through every part, and often startle him with the unexpected sight of marble obelisks, which in purity of appearance might pass for emblems of those who had " washed their robes and made them white in the blood of the Lamb." This beautiful and solemn burial place belongs to the city, which conveys small portions of it by deed in fee simple to families, but retains jurisdiction over the whole, and protects every work of art and every flower, down to the humblest blue violet, by adequate penalties and a vigilant police. Finally, a portion is reserved as a " Potter's Field," and it is interesting to see with what pains the poor in many cases have sought to decorate the graves of friends, which under other regulations would be left to sink beneath the surface and be seen no more.

Now, I hope you will not strike this hasty paragraph from my letter as of no interest to our readers. If they should not be interested in it, they ought to be; for there is no class of men who could do more to promote the establishment of such cemeteries, and certainly their refining, moral influence is imperatively needed, seeing how small is our veneration for the monuments and memory of the dead.

I had hoped to include in one letter all that I might wish to say of New York, but find I cannot without tiring out both our readers and yourselves. I shall therefore subscribe myself

Your obedient servant,

D.

### TRAVELING LETTERS FROM THE SENIOR EDITOR.

NUMBER FOUR.

GROS ISLE, 33 miles below Quebec, August 27.

*To my Colleagues:*

GENTLEMEN: Since I left Rochester, where my last was finished, I have seen and heard many things miscellaneous which I might embody in a letter; but presuming that our readers have been sufficiently dosed with them, and finding myself in the midst of an epidemic which strikingly sustains the old and universal association of words—famine and pestilence—I propose to let the past remain where it is, and devote myself to the present.

Quebec is the terminating point of steampacket navigation on the Saint Lawrence, but the Canadian government has procured the running, twice a week, of a small steamer from that city to the quarantine station. It is owned and directed by Mr. W. Stevenson, a respectable merchant of the city, who, h·aring of my desire to go down, politely offered me every facility. I am now on board about to return, as the boat will start as soon as the convalescents whom I see standing on the dock have been taken on board. On my way up, and afterwards, I propose to give our readers a rapid and superficial sketch of what I have seen here, and what I may hereafter see in Quebec and Montreal, though, as I have not seen our Journal for the last two months, I do not know but that you have already published enough on the subject.

Gros Isle is one of the endless succession of beautiful islands which adorn this noble river from Lake Ontario to the Gulf of St. Lawrence. Many of them consist of ancient *drift*, and have level surfaces which rise but a few feet above the water; but this is rocky and rugged, with heights of eighty or a hundred feet in its center, and hence the name given it by the early French voyageurs. Its breadth is less than a mile, with a length of nearly two. The black birch, white cedar and various kinds of pine overshadow and partly obscure its stony surface.

The quarantine station is on the southeast or right hand side, which in the approach presents three distinct groups of one-story board sheds, some of which are mere cottages, but others are from two to four hundred feet long. The lowest or eastern group is for the reception and temporary accommodation of immigrants in health; the next up the island is for the quarantine physician and a small detachment of troops from the garrison at Quebec; the third or western, more extensive than both the others, for the sick and their physicians, nurses, and a numerous body of carpenters engaged in the erection of additional houses to receive the hundreds who are still lodged in tents and marquees. The buildings of each group are whitewashed, and appear in pleasant contrast with the green slopes and tuberosities of the island in their rear. The harbor in front presents several ships at anchor, and two or three steamboats, with a neat and nearly finished dock projecting to a distance into the stream. When we were near the harbor, a gentleman, Mr. Patten, who resides in its neighborhood, and who had kindly directed my attention to different objects on our little voyage, called my eye to an Irish immigrant ship then passing us. On inspecting the group of passengers with a glass, I was surprised to find them so healthy in appearance; and when about to express myself to that effect, he discovered and announced that it was a ship from Bremen. Such is the difference between the German and Irish immigrants in health and personal condition.

A distant view of Gros Isle suggests a new and busy colony on a romantic shore, but a walk up the dock which leads directly to the hospital sheds most painfully dispels the pleasing illusion. As I approached them, the emaciated forms and haggard faces of convalescents—sauntering about, or crouched on the ground and rocks, or sitting underneath the eaves and on the piles of boards to be used for coffins—impressively told what might be expected within. Conducted by Mr. Patten, I passed through them without stopping till we reached the quarters of Dr. George M. Douglas, the health officer, who received me with much hospitality. I found him lame from a kind of hospital gangrene or slough which had attacked one of his feet, but, intent on his duties, he was bravely hopping about and answering a hundred questions or giving as many orders.

Taking me in his buggy, he drove to his office in the midst of the sick, where I was introduced to several of his assistants. They are chiefly young physicians of Quebec and other towns of Canada, employed by the government. One to whom I was introduced, although walking about, labored under fever, and yesterday I saw another at Quebec who had returned in the same condition. The number of assistants today is nine. Since the first of June, twenty-one or twenty-two have been employed. All except Dr. Douglas have experienced attacks of the fever, and three have died, one of whom was Dr. Frederick Cushing, formerly of the State of Maine. The exemption of Dr. Douglas is to be ascribed to his having already had the disease. After conversing awhile on its symptoms and treatment, Dr. Watt and Dr. Fenwick conducted me to their respective hospitals, embracing six or eight hundred patients, where I took such a *coup d'œil* of the sick as my limited time would permit, examining with some attention a considerable number in every stage of the disease. From a necessity which the Canadian government up to this time has been unable to avert, all the tents and sheds are crowded to such a degree that one can scarcely turn round among the sick. Men, women and children in all stages of the disease up to dissolution are huddled together, and lying in the same foul and infectious clothes with which they started from Ireland, and which no doubt they had worn without change for weeks or months before. The quarantine officers must not be blamed for this, since the means of classification and personal cleanliness are not within their reach. As to nursing, it is evidently in the lowest degree. Nearly all the nurses from Quebec have sickened, and the immigrants furnish but few from their own body. Their sympathies for each other are manifestly small—either never had an existence or have perished under the combined influence of famine and filth. Examples of the well members of a family refusing to wait on the sick are familiar to all the medical gentlemen, and a total indifference to the death of nearest relatives is witnessed every day. Following their remains to the grave, or in any manner assisting in their interment, is not thought of. But one idea seems to be present with them, that of getting up the river. A man who had recovered, on being asked by some one whether he was going to Montreal in the next steamboat, replied that he wished to do so, but was afraid his wife would not die in time. The family of a young woman who was ill *sent* to know how she was before they started; on being expostulated with, they said it was not worth while to stay any longer, as she would no doubt die. Mr. Barton, the apothecary of the hospital, who is now by my side going to Quebec on official business, confirms all that has been told me by others, and adds, as the result of his own observation throughout the summer, that the living seem more pleased than grieved by the death of their friends. My own limited opportunities suggest the same unwelcome conclusion; for I saw no aspect of sorrow, but a stolid indifference or inquisitive gazing at what might be passing around, both in the crowds of convalescents and in patients not very ill, who lay in the midst of the dying. It is painful to record this testimony against human nature, but we ought to know to what depths of degradation large masses of people may be sunk by superstition, ignorance, bad legislation, famine and fever. The interests of political economy, religion and medicine are equally involved in the contemplation of such revolting facts.

QUEBEC, August 28.

Before and since my trip to Gros Isle, I have visited the marine hospital of this city, (under the care of some of its most respectable physicians,) where a great number of seamen are down with the fever, and near which there are extensive sheds filled with sick immigrants. I have also been at the house of correction and in the Hôtel Dieu, where I saw cases, and at the private hospital of Dr. James o uglas and Dr. Racey, in Beauport, a village three miles from the city, where I saw still more. Many of the cases I examined with care, and held conversations more or less protracted with a number of the medical attendants, among whom I may mention

Dr. Morrin, Dr. Racey, Dr. J. Douglas, Dr. Clark, and Dr. Fremont, whom I may unite with the physicians of Gros Isle as the authors of what I am about to say on the history and treatment of the fever.

1. The pauper immigrants from Ireland are its chief victims; but it also affects the Irish pensioners, whose means must have kept them above the minimum of diet to which the former had been reduced by the famine; finally, it invades the officers and seamen of the ships which bring them over, and the physicians and nurses who wait upon them after their arrival. A great number die on the voyages, and many arrive ill; but it has been observed at Gros Isle that a large number are attacked soon after being landed. Others remain well and are sent on to Quebec, where a portion of them are taken down, while others escape till they reach Montreal or the towns above. When at Oswego, in the State of New York, on my way out, I saw a number of cases.

It affects men rather more than women, and adults more than children; hence it has increased the proportion of infants on the banks of the St. Lawrence to an unprecedented extent. I have already mentioned the mortality of the physicians at Gros Isle, a seventh of whom have died. In the marine hospital of Quebec, nine or ten of the old nurses have perished, and others are disabled, so that there is not now one on duty who was there before the fever was introduced. In the sheds both there and here, but especially there, the crowd of patients is so great that one, as I have said, can hardly turn round among them, and in several of them men, women and children are indiscriminately huddled together. As the government has not undertaken to furnish them with clothing, most of them lie in the foul and tattered garments which they wore during the voyage, and perhaps long before. Now, whether the disease is propagated by a gas developed chemically from the organic matter which surrounds them, or by a morbid aeriform secretion from their bodies, we are at no loss to account for the sickness of physicians and nurses. On the question of its spread beyond the sheds and hospitals I have sought for information. The medical gentlemen with whom I have conversed, without a single exception, are of opinion that it can be communicated by fomites, and cite instances of its appearing in families which had never communicated with any of the wards, but had employed those who had recovered from attacks. In the private hospital of Drs. Douglas and Racey, where there is cleanliness, free ventilation and ample space, very few of the attendants are attacked. On the whole, it appears to me that its mode of propagation should still be kept *sub judice*.

2. After seeing patients in every stage of the fever, and conversing with the gentlemen whom I have named, I may venture to give the following desultory account of its symptoms and progress:

Most of the cases are not seen in the beginning by physicians, and no reliable accounts can be got of them; but on the whole, the majority seem to sicken gradually; and in reference to those who had been greatly reduced by famine, this is perhaps always the case. There are, however, many examples of sudden and violent invasion, followed by a malignant development, and death in a few days. In no instance does the chill become very intense, though it may be protracted, nor is the arterial reaction high. In some cases the latter, in fact, never manifests itself—the vital forces being inadequate to a rally. The pulse is never tense, and in the highest reaction always easily compressible; its frequency is increased, but not to a remarkable degree; it often becomes almost imperceptible in those who recover. From the beginning, the primæ viæ are more or less, but variously disordered. In some there is nausea and vomiting; in all, loss of appetite, with thirst. Some are costive in the forming stage, and even throughout the fever; in others there is a *precursory* diarrhea; in the majority, a *supervening* diarrhea or actual dysentery. I could not ascertain that there is generally a superabundant excretion of bile. The tongue at the onset is always covered with white fur, through which the red papillæ sometimes show themselves; in a part, the edges and tip of the organ show some unnatural redness, but in the greater number the natural color is not exalted, but even reduced, so that the white fur seems to shoot out of a pallid membrane. At the same time the organ becomes broader and flatter, loses its elasticity, and receives indentations from the teeth, on which I seldom saw any sordes. Its moisture continues in a remarkable degree; it may be reduced, but not to the point of dryness; and the whiteness of the fur endures to a period equally late. The dry, contracted, mahogany tongue of genuine typhus often appears, it is true; but in numerous instances the moist and pale state of the organ continues up to the time of dissolution. The usual inequality of heat between the upper and lower parts of the body is common. I saw many patients in which the latter were cold, and some in which the former were decidedly hot; but great development of caloric is not, I think, a constant phenomenon. Delirium is more prevalent than coma; many patients during the night, when it is greatest, are restless and even locomotive, becoming the next day composed and of sound mind. Somnolency did not appear to me to be a conspicuous symptom. Headache is often present. Of the red and dull eye I saw much less than I had expected. A circumscribed flush of the cheek is frequent, but not universal. A bilious tinge of the visage occasionally shows itself. Subsultus tendinum is comparatively rare. I saw many who seemed to be almost in *articulo mortis*, and yet showed little or none of that symptom.

The skin shows various kinds of maculæ. In a few, genuine rose-colored spots show themselves, but very soon assume a darker color. In the majority, the spots are purple from their first appearance, and of every size from ordinary petechiæ up to diffused echymoses, often bearing a close resemblance to post-mortem hyperæmias. In some cases the spots are hard like whelks, and the seat of a sensation which leads the patient to scratch them, whereupon ulcers follow, which occasionally assume a sloughing character. Hemorrhages from the nose are somewhat common, from the bowels and skin not quite so frequent; nevertheless all the medical gentlemen have had cases of well marked purpura hæmorrhagica mixed up with the fever cases; and it may be safely affirmed that in these immigrants the blood, under the influence of a reduced or unhealthy diet, has become signally deteriorated.

When the fever assumes a protracted form, anasarcous infiltrations into the cellular tissue of the lower extremities or the face frequently take place. Suppurations, in addition to those of the skin just mentioned, are common. Those about the back and hips may be ascribed to pressure; but others, occurring in glands, must be referred to the fever. Of these organs, the parotids suffer oftener than all the rest, and the discharge of pus when they suppurate, is copious. Such cases generally end well.

A supervening bronchial or pulmonary affection is, on the other hand, ominous, and as it frequently occurs, may be considered one of the modes in which the fever comes to a fatal termination.

But of all the secondary affections, that of the bowels is most frequent and fatal, though death may not occur for a considerable time after the febrile period has expired. This intestinal disorder seems to be a sort of mixed up diarrhea and dysentery, under which the patient loses the original febrile symptoms, and, becoming extremely emaciated, gradually sinks. In some instances this affection sets in during the fever; in others it is excited in the period of convalescence by irregularities of diet; in all it is an ugly, obstinate and unmanageable *addendum*. In the months of June and July it was much less frequent than at the present time, when so large a proportion of the patients labor under it as almost to constitute it a new act in the melancholy drama.

I have mentioned the nocturnal delirium of some patients, indicating an exacerbation at night, and may add to this evidence of periodicity that in a few cases there has been a diurnal recurrence of the initial chilliness; the general character of the fever, however, is continued. I have spoken of cases which prove fatal in three or four days; they are few in number, and the common duration is from two to three weeks, always excepting those which merge in diarrhea or dysentery, when the end is quite indefinite.

When death is the consequence of cerebral, pulmonary, hepatic or intestinal concentration, the reason of its occurrence is intelligible enough; but the majority do not seem to die from these lesions, and the cause of their dissolution is, *prima facie*, rather obscure. In every ward that I visited I

was surprised at the small amount of visible manifestation of dangerous disease, and more than once was prompted to say to the medical gentlemen, "I can't see why so many of your patients die." In wards from which many corpses were daily carried out, there would be but few who did not look at and after us, put out their moist tongues with facility, and make known their wants; yet many such patients die soon afterwards; others die when the physician has pronounced them convalescent; others after they have risen and dressed themselves, and crawled into the open air. Such deaths cannot be regarded as the effect of any particular organic lesion, but of a state of exhaustion or collapse, bearing some resemblance to the third stage of yellow fever, or the recurring chill of a malignant intermittent in the South-west, but more, perhaps, to the fatal stage of epidemic cholera.

After this imperfect, but I believe not unfaithful sketch of the history of this fever, I must proceed to its cure:

3. It is a maxim with the physicians of Gros Isle and Quebec, that a great amount of medication is inadmissible in this malady. Scarcely a patient is ever brought to them while the fever is still in its forming stage, so that there are but few opportunities of knowing whether art could arrest it in that stage. Once established in the system, the general opinion is that it cannot be cut short. The treatment then is merely palliative and corroborative.

By all the physicians in almost every case the lancet is repudiated, even when the patients are commanders of ships, and seamen, in whom famine had not preceded the attack. Local bleeding is almost as little employed, the majority of the physicians preferring counterirritants when the brain or the lungs are affected.

Emetics are prescribed by some, who speak well of their effects; but others think they predispose to congestions of the brain. It has not been observed by any that they break up the disease.

All employ cathartics, but the kinds and the degrees of their administration present considerable variety, into which, however, I shall not enter, as drastic or long continued purging is condemned by the whole,

Of sweating I can say nothing, for the patients generally are placed under such circumstances as preclude a resort to it.

A standard or standing febrifuge mixture at Gros Isle, as given me by the apothecary, Mr. Barton, is the following. ℞. Sod. et pot. tart. ℥ij; liq. ammon. acet. ℥jss; spt. ether. nit. ℥ss; aquæ commun. ℥xij. Misce. Another is composed of the camphorated mixture, tincture of hyoscyamus and tartar emetic.

Some of the medical gentlemen attach but little value to this class of medicines, and rely, during the more acute stages of the fever, chiefly on free dilution, but advise against the addition of acids, as likely to irritate the bowels into diarrhea.

Opium is not in much favor in any stage of the fever. At a comparatively early period some physicians commence the administration of wine and aliment—a practice condemned by others, as dangerous to the brain; but all concur in this, that sooner or later, and sometimes quite early, there comes a pathological condition which demands prompt and energetic stimulation. In this resort a choice of means is not to be neglected. Of the whole materia medica, camphor is the most reliable. It is given in doses of ten, twenty, or even thirty grains, and often arrests the sudden sinking of the powers of life, and determines a speedy recovery. At the same time, alcoholic stimulants, sulphate of quinia, food and sinapisms are employed.

For the diarrhea and dysentery, the cretaceous mixture, hydrarg. cum creta, astringents, and diluted nitric acid, with laudanum, are the usual prescriptions.

MONTREAL, September 3.

A large number are sick in the sheds at this place, attacked, of course, after they had left the quarantine ground. A great proportion of them are said to have dysentery. The deaths are numerous. The fever here is by no means confined to immigrants, but has invaded the city and added greatly to its ordinary summer mortality. Its victims, however, are largely of that class which, living near the wharves, received and mingled with the immigrants before sheds were provided for their reception. In conversing with several of the most intelligent physicians here, I find the treatment to be substantially the same as at Gros Isle and Quebec; but latterly, as Dr. Badgely informed me, increasing reliance is placed on nitric acid. He uses the following formula: ℞. Acid. nitr. ʒj; alcohol, ℥iv; aquæ, ℥iv. Misce. An ounce is to be given every hour, beginning early in the disease and without much preparation of the system. Under its administration, he and other gentlemen have, as he assured me, seen the pulse rapidly reduced in frequency, with a corresponding abatement of all the febrile symptoms.

When at Gros Isle, I inquired as to *post-mortem* inspections, and could hear of only two. They were made by Dr. Watt, who found the liver engorged, and, as he believed, fatty. The state of the organs convinced him that the one mentioned suffered more than any other, which led him to prescribe purges of calomel and gamboge. In Quebec I could not learn that any dissections had been made. In this city Dr. Fraser has published, in the July number of Dr. Hall's valuable "British American Journal of Medical and Physical Sciences," a short paper, in which he says:

"The morbid appearances found on dissection are venous congestion, with effusion of serum on the surface, in the ventricles, and base of the brain, but no trace of active inflammation. When the case has been complicated with bronchitis, I have found the bronchial mucous membrane throughout tumid, swollen, highly vascular, and containing much mucus; the vascularity extending to the submucous tissue, with congestion and partial hepatization of portions of the lungs. When diarrhea has existed, the small intestines, especially the lower portion of the ileum, has presented the appearance of active congestion of its mucous coat, which was slightly thickened without being softened; some patches had the appearance of sanguineous extravasation, not unlike the maculæ observed on the skin. When the patient had a jaundiced appearance, a common occurrence in this epidemic, I have found the liver enlarged from congestion, presenting a bloody and bilious appearance when cut into, and the gall-bladder distended with inspissated bile thick enough to maintain its form when deprived of its covering. When there has been only a slight bilious tinge of the skin and conjunctivæ, the liver presented the same appearance in a less degree, the bile in the gall-bladder being about the consistence › treacle."

Dr. Frazer does not tell us how many autopsies he had made, and we cannot but regret that so little has been done where the opportunities are so great. It may be given in extenuation, however, that the physicians in attendance are overworked, and that many of them have been ill with the fever.

I have no time to give you the statistics of this disease, had I been able to collect full data. The number of deaths in proportion to that of the cases never will be known, as nearly every form of disease in the immigrants goes very much under one name. I may say in general terms, the mortality has been and still continues to be very great. Take the following as specimens: At Gros Isle, for the week preceding the 24th of August, the number of patients averaged more than 2000, and the deaths amounted to 288. In the marine and emigrant hospital at Quebec, the average for the week ending the 21st of August was about 850, the deaths 166; to which might be added 34 said to have died of dysentery, a sequel of the fever. The Montreal returns would be about the same, and present in the aggregate about 100 deaths a day at these places. At Gros Isle alone, up to the 20th of August, the deaths had amounted to 2116. The disease had then prevailed about three months. A portion of them, however, have resulted from smallpox, which has prevailed to a considerable extent on the Island, and more or less, I believe, in Quebec and Montreal—certainly in the latter, where I saw it in the general hospital.

The British government has lately made an effort to arrest the fever by disinfecting agents. Dr. Stratton, of the royal navy, but sojourning in Upper Canada, has been commissioned to this enterprise, and arrived in

Quebec while I was there. He was advised that two agents would be forwarded to him. One proved, on opening the package, to be Sir William Burnett's patent disinfecting fluid; the other, a small bale of dresses for a lady! which had been forwarded from Halifax (by mistake) instead of Ledoyen's disinfecting fluid. The former—of which Dr. Stratton kindly presented me with a small vial, which I carry in my trunk as an amulet—is said by the medical gentlemen of this quarter to be a solution of the chloride of zinc; the latter, according to Dr. Hall, is a liquor of nitrate of lead. When I left Quebec, arrangements were making for experiments in the marine hospital, but its physicians seemed to think lightly of the practical value of such measures. When I was at Gros Isle, Dr. Douglas, the quarantine physician, sagaciously remarked that the proper place for such experiments is an emigrant ship on her voyage.

I shall ascend the St. Lawrence from this place to Kingston and Toronto, rëentering our own country at Buffalo. Whether I shall be able to send you another letter is uncertain, as I am admonished by a slight tinge of yellow in the woods that autumn is at hand, and that the opening duties of next winter are not, in time, very far off.

Respectfully, your colleague, D.

